# Development and usability testing of a Web-based decision aid for families of patients receiving prolonged mechanical ventilation

**DOI:** 10.1186/s13613-015-0045-0

**Published:** 2015-03-25

**Authors:** Christopher E Cox, Nicholas G Wysham, Brenda Walton, Derek Jones, Brian Cass, Maria Tobin, Mattias Jonsson, Jeremy M Kahn, Douglas B White, Catherine L Hough, Carmen L Lewis, Shannon S Carson

**Affiliations:** Department of Medicine, Division of Pulmonary and Critical Care Medicine, Duke University, Durham, NC 27710 USA; Program to Support People and Enhance Recovery, Duke University, Durham, NC USA; Center for Learning Health Care, Duke Clinical Research Institute, Durham, NC USA; Information Technologist, Sheps Center for Health Services Research, University of North Carolina at Chapel Hill, Chapel Hill, NC USA; Information Technologist, Lineberger Comprehensive Cancer Center, University of North Carolina at Chapel Hill, Chapel Hill, NC USA; Department of Critical Care Medicine, University of Pittsburgh School of Medicine, Pittsburgh, PA USA; Department of Medicine, Division of Pulmonary and Critical Care Medicine, University of Washington, Seattle, WA USA; Department of Medicine, Division of General Internal Medicine, University of Colorado, Denver, CO USA; Department of Medicine, Division of Pulmonary and Critical Care Medicine, University of North Carolina at Chapel Hill, Chapel Hill, NC USA

**Keywords:** Critical illness, Usability, Patient reported outcomes, Patient-centeredness, Surrogate decision making, Decision aid, Decision support, Chronic critical illness, Prolonged mechanical ventilation

## Abstract

**Background:**

Web-based decision aids are increasingly important in medical research and clinical care. However, few have been studied in an intensive care unit setting. The objectives of this study were to develop a Web-based decision aid for family members of patients receiving prolonged mechanical ventilation and to evaluate its usability and acceptability.

**Methods:**

Using an iterative process involving 48 critical illness survivors, family surrogate decision makers, and intensivists, we developed a Web-based decision aid addressing goals of care preferences for surrogate decision makers of patients with prolonged mechanical ventilation that could be either administered by study staff or completed independently by family members (Development Phase). After piloting the decision aid among 13 surrogate decision makers and seven intensivists, we assessed the decision aid’s usability in the Evaluation Phase among a cohort of 30 surrogate decision makers using the Systems Usability Scale (SUS). Acceptability was assessed using measures of satisfaction and preference for electronic Collaborative Decision Support (eCODES) versus the original printed decision aid.

**Results:**

The final decision aid, termed ‘electronic Collaborative Decision Support’, provides a framework for shared decision making, elicits relevant values and preferences, incorporates clinical data to personalize prognostic estimates generated from the ProVent prediction model, generates a printable document summarizing the user’s interaction with the decision aid, and can digitally archive each user session. Usability was excellent (mean SUS, 80 ± 10) overall, but lower among those 56 years and older (73 ± 7) versus those who were younger (84 ± 9); *p* = 0.03. A total of 93% of users reported a preference for electronic versus printed versions.

**Conclusions:**

The Web-based decision aid for ICU surrogate decision makers can facilitate highly individualized information sharing with excellent usability and acceptability. Decision aids that employ an electronic format such as eCODES represent a strategy that could enhance patient-clinician collaboration and decision making quality in intensive care.

**Electronic supplementary material:**

The online version of this article (doi:10.1186/s13613-015-0045-0) contains supplementary material, which is available to authorized users.

## Background

Decision making in the setting of critical illness can be difficult for patients, families, and clinicians alike [1]. When such decision making is not collaborative or is perceived to be inadequate, the patient centeredness of care suffers and the decision makers themselves may experience subsequent psychological distress [2]. Shared decision making, a dynamic process of consensus building where responsibility for making decisions reflecting the alignment of values and choice is shared between health care team and the patient or patient’s surrogate, is a strategy increasingly utilized to address these problems [3,4]. Although shared decision making is the recommended strategy for navigating choices in complex treatment plans [4,5], evidence suggests that it remains insufficiently adopted [6].

Decision aids are printed, electronic, or video programs that can promote shared decision making and improve decision making quality [7]. We recently developed a printed decision aid to assist the process of shared decision making among surrogate decision makers of critically ill patients and their providers facing a decision about the provision of prolonged life support [8]. This decision aid showed evidence of feasibility and acceptability, while also demonstrating plausible impact on reducing surrogate-clinician discordance about prognosis, psychological distress, and decisional conflict. However, printed decision aids have a number of limitations. They cannot be easily individualized, they are not interactive, require cumbersome manual data entry after completion, and they may be perceived as less engaging than other formats.

To address these problems, we sought to expand a brief printed prolonged mechanical ventilation decision aid to a fully electronic, multi-function version that we termed ‘electronic Collaborative Decision Support’ (eCODES). We studied whether or not eCODES was actually usable and acceptable by decision makers for use in clinical care and research. Here, we report the results of this effort as well as the lessons we learned during the developmental process that could assist others engaging in similar future projects.

## Methods

**Figure 1:**
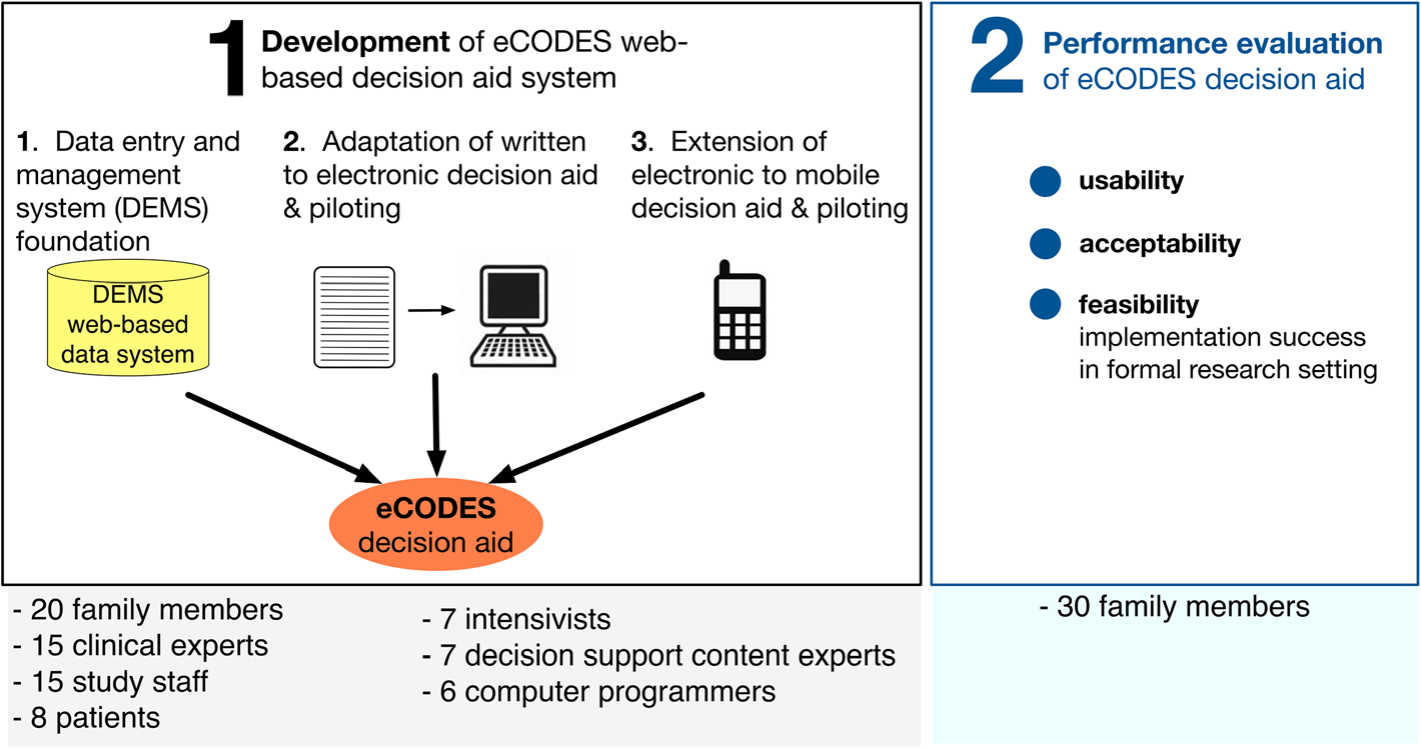
**Overview of study.** The *development* of eCODES, a Web-based prolonged mechanical ventilation decision aid, included extension of a data entry and management system to include a study staff-directed electronic decision aid adapted from a printed version. Development also included enhancing eCODES with a mobile functionality, allowing users to view it at their convenience in locations outside a hospital setting. The *performance evaluation* phase included a formal assessment of usability, acceptability, and feasibility.

This study includes two components, a *Development Phase* and an *Evaluation Phase* (Figure 1), completed over a period of nearly 3 years. In the Development Phase, we adapted our previously validated printed prolonged mechanical ventilation decision aid to create a Web-based version (eCODES). Family members who piloted eCODES provided written informed consent. In the Evaluation Phase, we measured eCODES’ usability, acceptability, and feasibility among family members of ICU patients. We conducted this phase under a waiver of consent granted by the Duke University Institutional Review Board because we did not record any personal information, only gender and age range. The timeline of the entire decision aid project including both the printed and Web-based versions is shown in Additional file 1: Figure S1 (“Evolution and development of web-based decision aid).

### Phase 1: Development of eCODES

#### Original printed decision aid

The original prolonged mechanical ventilation decision aid for surrogate decision makers was developed in accordance with the consensus guidelines and piloted in 2009 to 2011 as previously described [8,9]. In short, we developed a brief printed decision aid that framed the decision about prolonged life support preferences as a spectrum ranging from aggressive, survival-focused treatment to care focused purely on symptoms. By helping to place patients along this spectrum, the decision aid provided a concrete framework on which a facilitated discussion between surrogate decision makers and clinicians could take place.

#### Version 1 of the Web-based decision aid

The initial Web-based version of the decision aid adapted the printed version nearly page for page, but added a password-protected area that allowed the research team to enter five clinical variables measured on the tenth day of ventilation to populate the day 10 version of the prolonged mechanical ventilation (ProVent) score for prediction of 1-year mortality: age, use of vasopressors, requirement for hemodialysis, platelet count, and trauma status [10]. Our choice to use the ProVent model in the decision aid defined prolonged mechanical ventilation as 10 or more days of ventilation. In this version of the decision aid, prognosis was displayed as a bar graph in early mockups. This version met 11 of the 13 International Patient Decision Aid Standards consensus criteria, two effectiveness criteria are still under evaluation [9].

#### Version 2 of the Web-based decision aid

We then worked with a consultant computer programmer and graphic design firm to develop a more engaging version of the decision aid that could be integrated into a secure data management system. We created storyboards, page outlines, computational infrastructure for entry of variables necessary for the ProVent model, and a better graphical display of prognosis. We added more extensive content on the decision itself, about common ICU therapies such as mechanical ventilation, and on how to understand prognostic estimates. The final version was a section-tabbed, interactive program that summarized the user’s interactions and responses in a single PDF document that could be printed from the website, free of protected health information (PHI).

#### Version 3 of the Web-based decision aid (eCODES)

**Figure 2:**
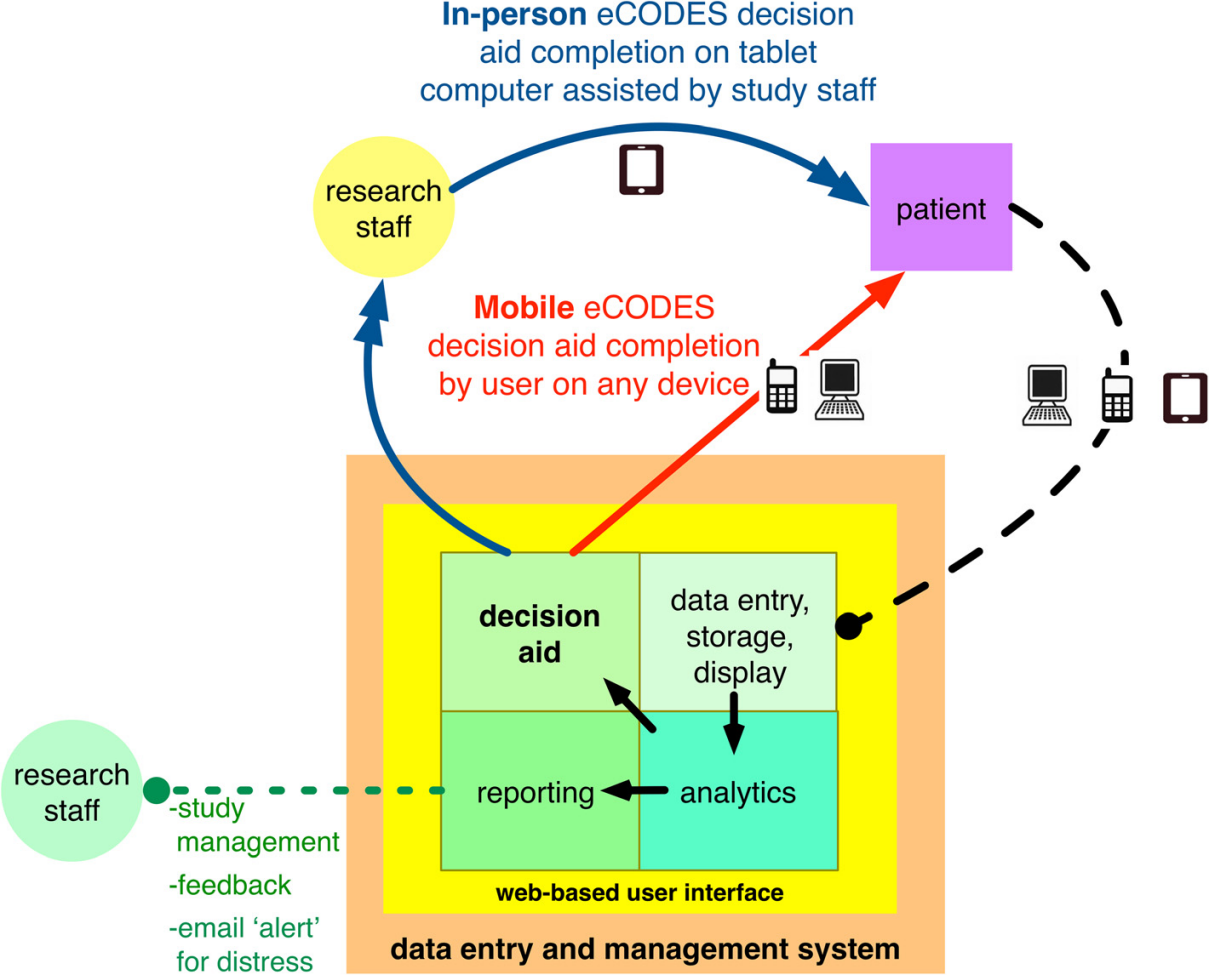
**Orientation of eCODES within an electronic data entry and management system.** eCODES resides on a secure server and is accessed via a Web user interface. eCODES can be administered by study staff in person or completed securely by surrogate decision makers on a separate device.

The programming code for the decision aid v2 served as the foundation for the third and final version of the Web-based decision aid, eCODES. eCODES included an improved help function as well as an optional tutorial on using tablet computers. The display of prognostic data was a major focus of revision in this version. In particular, we programmed a page flow to try to display potentially serious prognostic estimates as sensitively and deliberately as possible. eCODES required users to touch different screen areas corresponding to explanatory text in order to move forward (to avoid ‘blind clicking’ to a page they were unprepared to see) and to also warn them about the information they were about to view. eCODES was first designed to be administered in person on a tablet computer by either a clinical or research team. Soon thereafter, in response to family requests, we added a secure, password-protected, HIPAA-compliant, PHI-free version that could be viewed outside the hospital setting. We integrated eCODES into an equally secure Web-based data entry and management system (see Figure 2 and Additional file 2, “Security standards of study data system used to present eCODES.”) built specifically for the randomized clinical trial designed to systematically test the impact of the decision aid (NCT01751061).

### Phase 2: Evaluation of eCODES

#### Design and participants

For eCODES formal user testing, we recruited a convenience sample of 30 family members of ICU patients from the medical ICU waiting room at Duke University in six separate sessions between 2 September 2014 and 12 September 2014. The participants were asked to read a standard clinical scenario, perform an eCODES session, and then complete a short interview.

#### Clinical scenario and eCODES session

Each participant received an identical one-page printed document (shown in Additional file 3, “Instructions for usability testing.”). It described a hypothetical situation in which their 60-year-old loved one had received 10 days of mechanical ventilation and had just initiated hemodialysis. The document also emphasized that their task was to decide what to do next with life support according to the goals of treatment options specified in the original printed prolonged mechanical ventilation decision aid (treatment with comfort as the main goal, aggressive care with survival as the main goal, or treatment that aimed for survival but without prolonged life support) [8]. After reading the document, each participant viewed a tablet computer-based eCODES session that was pre-programmed to display prognosis mirroring the clinical scenario (approximately 70% likelihood of death within 1 year). They were not coached further at this point.

#### Interview and study outcomes measures

Immediately after finishing the eCODES session, the participants completed a short-printed questionnaire to assess the decision aid’s *usability* and *acceptability*. Usability describes the quality of a user’s experience with software or an information technology, taking into account their own needs, values, abilities, and limitations [11]. We used a well-validated, industry standard to measure usability, the Systems Usability Scale (SUS). The SUS is a 10-item, Likert-scaled (strongly agree, agree, neutral, disagree, and strongly disagree) measure with scores ranging from 0 to 100 [12]. The SUS items address to what extent users perceive the program as complex, cumbersome, easy to use, requiring support of a technical expert to complete, has integrated functions, has consistency, is learned quickly and confidently, or requires significant learning prior to use. The target SUS score was >70, representing ‘good’ usability [13]. Guided by the recommendations of Usability.gov, we also assessed usability domains using Likert-scaled items addressing learnability (how easy it is to accomplish tasks the first time), success rate (login, question completion, viewing all necessary pages), possible barriers, efficiency (task time) [11,14]. We used two sample *t* tests to compare SUS scores by age range. We assessed *acceptability* using measures of satisfaction (overall, layout, content, and preference for printed vs. Web-based version) assessed with a 5-point Likert scale; we targeted a satisfaction level of 80% for ‘good’ acceptability. The participants provided written open-ended feedback to highlight the eCODES elements they most liked and disliked as additional measures of acceptability. Study staff assessed error rates by comparison of completed eCODES episodes and any translational errors recorded in the study data system. While not the primary aim of this study, we assessed *feasibility* of eCODES by the rater of successful sessions completed (in person and mobile versions) to date among persons enrolled in the parent randomized controlled trial.

## Results

### Phase 1: Development of eCODES

#### Development

This intensive process of storyboarding, programming, evaluating, and revising across three versions of the decision aid included 20 family members, 15 surrogate decision makers of ICU survivors, 15 clinical research coordinators with extensive experience interviewing patients and families in an ICU research setting, eight ICU survivors who received mechanical ventilation, seven intensivists, seven decision support content experts, and six programmers (Figure 1). In particular, study staff spent many hours testing the decision aid repeatedly as if they were surrogate decision makers, with in-depth examination for potential problems.

The final version of the decision aid, eCODES, included several key characteristics that represented comparative improvements to the printed version. First, it was an engaging, Web-based, graphics-heavy program that could be either administered on a touch screen computer by study staff or viewed on Web-enabled devices outside the hospital setting (see Additional file 4: Figure S2 comparing print to electronic versions). Second, we greatly improved the process by which potentially surprising prognostic information was revealed sensitively for the viewer. We used a combination of techniques to do this which slowed the pace of viewing, did not permit ‘rapid clicking’ through pages by requiring specific screen areas be touched, and asking the user if they were ready to see serious information. Third, the response choices for questions were more intuitively laid out - specifically for items allowing weighting on linear scales (e.g., anchors with ‘most’ and ‘least’). And finally, eCODES included a summary PDF document that was generated at the conclusion of the decision aid session for the purpose of providing a family meeting discussion focus for surrogate decision makers and clinicians.

#### Piloting eCODES in clinical research settings

We piloted eCODES in both study staff-administered and self-completed (mobile) versions at Duke University, the University of North Carolina, and the University of Pittsburgh among 13 surrogate decision makers and seven intensivists. Open-ended participant feedback was uniformly satisfactory, and all participants were able to complete the decision aid. Data transfer accuracy between the decision aid and the study data system was hand-verified to be error-free.

#### Phase 2: Evaluation of eCODES

A total of 30 participants evaluated the final online decision aid and completed the study interview, 20 of whom (67%) were female. Twelve surrogates (40%) were 45 years of age or younger, seven (23%) were ages 46 to 55, and 11 (37%) were 56 years and older. Users generally spent 15 to 20 min completing the decision aid program.

The mean SUS score was 80 ± 10 overall, a rating considered to reflect ‘excellent’ usability [15]. Mean SUS scores were highest among those <45 years (84 ± 9) and lowest among those 66 years of age and older (67 ± 8) (Table 1). Statistically significant differences in mean SUS score by age were observed between those ≤55 years (84 ± 9) and those 56 years and older (73 ± 7); *p* = 0.03. However, no statistically significant differences were observed within younger participants (<45 vs. 46 to 55) or older participants (56 to 65 vs. 66 and older); *p* > 0.05.

**Table 1:** **Usability attributes of eCODES by age group**

	**≤45** ***n*** **= 12**	**46 to 55** ***n*** **= 7**	**56 to 65** ***n*** **= 6**	**≥66** ***n*** **= 5**	**Total** ***n*** **= 30**
Selected Systems Usability Scale total scores					
Mean (SD)	84 (9)	85 (11)	75 (7)	68 (8)	80 (10)
Median (IQR)	83 (80, 88)	95 (80, 95)	74 (70, 78)	68 (60, 75)	80 (73, 88)
Range	70 to 95	68 to 95	70 to 88	60 to 75	55 to 95
Systems Usability Scale items*					
I think that I would like to use this program if I had a loved one in the ICU	11 (92%)	7 (100%)	5 (83%)	5 (100%)	28 (93%)
I thought the program was too complex	0 (0%)	0 (0%)	0 (0%)	0 (0%)	0 (0%)
I thought the program was easy to use	11 (92%)	7 (100%)	5 (83%)	4 (80%)	27 (90%)
I think that I would need the support of a technical person to be able to use the program.	0 (0%)	0 (0%)	0 (0%)	1 (20%)	1 (3%)
I found the information and questions in the program were well integrated	10 (%)	7 (100%)	6 (100%)	5 (100%)	28 (93%)
I thought there was too much inconsistency in the program.	0 (0%)	0 (0%)	0 (0%)	1 (20%)	1 (3%)
I would imagine that most people would learn to use this program very quickly	12 (100%)	7 (100%)	6 (100%)	5 (100%)	29 (97%)
I found the program very cumbersome to use.	0 (0%)	0 (0%)	0 (0%)	0 (0%)	0 (0%)
I felt very confident using the program	11 (92%)	7 (100%)	6 (100%)	4 (80%)	28 (93%)
I needed to learn a lot of things before I could get going with this program.	0 (0%)	0 (0%)	0 (0%)	0 (0%)	0 (0%)
I was able to:**					
Answer the questions in the program	12 (100%)	7 (100%)	6 (100%)	4 (80%)	29 (97%)
Complete the computer program	12 (100%)	7 (100%)	6 (100%)	5 (100%)	30 (100%)
I was satisfied with:***					
The computer program overall	12 (100%)	7 (100%)	5 (83%)	4 (80%)	28 (93%)
How easy it was to use the program	12 (100%)	7 (100%)	6 (100%)	4 (80%)	29 (97%)
The layout of the program	11 (92%)	7 (100%)	6 (100%)	4 (80%)	29 (97%)
The instructions	12 (100%)	7 (100%)	6 (100%)	4 (80%)	29 (97%)
Prefer eCODES to printed version of decision aid	11 (92%)	7 (100%)	6 (100%)	4 (80%)	29 (97%)

*n (%) who reported ‘strongly agree’ or ‘agree’. **n (%) who were able to perform the task. ***n (%) who were ‘very satisfied’ or ‘satisfied’.

While older participants’ SUS score was lower than those of younger participants, 10/11 (91%) of those 56 years and older stated that they would prefer Web-based to printed versions decision aid. Acceptability was further supported by the fact that over 90% of respondents reported satisfaction with the program overall, its ease of use, its layout, and the instructions. Open-ended written user comments were generally positive as well (Table 2). Representative written responses to the question, ‘What did you like most about the program?’ included ‘easy to use’, ‘user-friendly’, ‘informative’, and ‘interactive’. Several respondents’ responses suggested that eCODES helped to clarify decisional roles, to focus attention on patients’ values, and to show clinicians how family decision makers were leaning in their decision.

**Table 2:** **Open ended written feedback for eCODES by age group**

	**45 and younger** ***n*** **= 12**	**46 to 55** ***n*** **= 7**	**56 to 65** ***n*** **= 6**	**66 and older** ***n*** **= 5**
What did you like most about the eCODES program?	• Easy information particularly if you have no experience in this situation		• iPad is a familiar platform	• Easy to use
	• Easy to use		• (it asked) good questions	• Informative
	• It puts it in black and white		• (helps) reinforce the decision	• Information is easy to use and helpful
	• It focuses the question at hand on the patient		• Wording is simple	• Gives another data point
	• User friendly		• Interactive	
	• Self-explanatory			
	• How it broke the thought process down			
	• [Gives] explanations for different treatments			
	• I liked how patient and family centered it was			
	• Informative			
	• It was interactive			
	• You could get good information for your loved one			
What did you dislike most about the eCODES program?	• A sensitive topic		• Doesn’t make decision for you	• Simplistic
	• I don’t like touch screens		• Back/forth buttons have a delay	
			• I am an impatient person with computers	
How could the eCODES program be improved?	• Would like even more information about prognosis			• Make [the information] more complex
	• Make forward button more obvious			
	• Even more illustrations			
	• Make more options focusing on each specific patient’s case			

Seven (23%) participants provided written feedback about specific dislikes. Some critiques addressed technical issues such as touch screen capabilities, delays after pressing back/forth buttons, or a general dislike for electronic interfaces. Content critiques were also noted such as by one user who worried that prolonged life support was ‘a sensitive topic’ while another described eCODES and its sixth grade reading level as ‘simplistic’. Suggestions for improvement mapped to the above complaints, asking for improvements in navigation, more illustrations, and more information and detail.

Finally, hand checking of completed decision aid episodes, searchable through our study data entry and management system, showed that all eCODES sessions were complete and produced no data transmission errors. Further, the content of the summary PDFs produced by eCODES was verified to be harmonious with the actual data entered by the participants. To date in our ongoing clinical trial of eCODES [16], all surrogate decision makers have completed the decision aid successfully (215 in person and 37 on either a mobile device or home computer) - observations supporting the feasibility of using eCODES in a research setting.

## Discussion

We developed eCODES, a Web-based decision aid for surrogate decision makers of patients receiving prolonged mechanical ventilation. eCODES was highly usable and acceptable to the target audience based on a standard rating tool. Overall, 93% of users stated that they preferred eCODES to a printed decision aid.

Decision aids are becoming increasingly common in a range of diverse conditions and clinical settings because they can reduce decisional conflict, improve patient-clinician communication, improve the alignment of values and choice, and reduce the use of low value interventions [7]. The time-sensitive nature and frequently the suboptimal conduct of decision-making in the ICU highlight the necessity of tools to improve the efficiency and quality of the shared decision-making process [2,6,17]. Web-based or electronic decision aids have the potential to improve the cost of disseminating these tools and improve their efficacy by allowing customization of content or prognostic information based on patient’s medical, demographic, educational, cultural, or religious characteristics.

eCODES is one of the first decision aids specifically directed towards surrogate decision makers of ICU patients. This role has been associated with significant conflict and personal distress, including a high incidence of PTSD-like symptoms [18]. eCODES incorporates many of the evidence-based principles to improve the quality of shared decision making and communication that may mitigate this impact for surrogate decision makers in the ICU - namely, provision of clear information, assistance with eliciting and considering patient (as opposed to surrogate) values, and focusing on collaboration. Another valuable aspect of eCODES is its ability to export a printed distillation of the user’s experience that can be used as a center point for a family-clinician meeting, thus emphasizing collaboration and information sharing.

The development of eCODES was motivated largely by family member feedback; the decision aid’s high usability likely reflects their involvement in the iterative design process. As health care system participants increasingly trust and utilize digital health information, Web-based tools like eCODES will become increasingly common and used [19,20]. Electronic decision aids have the potential to integrate with electronic health records (EHRs), perhaps being prompted automatically in patient- and family-facing portals when relevant clinical situations arise. The challenge will be to help patients and families interpret and personalize complex health information to best meet their needs and abilities. One relevant example in eCODES was the way we carefully constructed the presentation of individualized prognostic information to deliver this content in an easily understood and respectful manner. Because prolonged mechanical ventilation prognosis is often poor, our best efforts at presenting difficult information with tact, respect, and sensitivity may not be successful among all users - an area that requires further iterative revision.

This study also highlights the difficulty and resulting expense of developing a simple, usable decision aid for a complex use scenario that appeals to diverse groups of users. One can only imagine the resources needed to produce similar decision aids for all potential conditions. This reality highlights the need for a more comprehensive ‘universal’ approach to decision support that includes a common platform to standardize processes, a method for measuring and incorporating decisional outcomes feedback into decision making (e.g., electronic patient reported outcomes [PROs]), an approach to personalizing the patient/user experience, and a learning health system-framed predictive analytics function informed by PRO and EHR system data [21-23]. While it may seem futuristic to those who are accustomed to the frustratingly fragmented clinical health informatics systems, it is telling that the electronic decision aid capability was anticipated and requested unprompted by our test users.

We recognize several important limitations. First, usability lacks a single accepted metric. We tried to address this by assessing multiple quantitative dimensions of usability and also eliciting open-ended feedback. Secondly, our sample size was relatively small and therefore may not reflect the values and experiences of all populations. We aimed to address this by purposefully sampling diverse age ranges, ethnicities, and ICU locations, though. Third, we found somewhat lower satisfaction and usability among those with older age - a group known to use mobile technology less frequently than younger groups [24]. While age-related disparities in adoption of mobile information technology will likely recede with time, we recognize that user interaction improvements could be important for the elderly. It is estimated that while 86% of adults have Internet access, many of lower socioeconomic status (a variable we did not measure) may have unique information technology needs [24]. Finally, we have described the development and user testing of eCODES in a hypothetical-use situation to attempt to focus users’ attention on the decision aid and to control somewhat for patient-, personal-, and clinician-related effects that could cloud objectivity in a real-world scenario. The clinical efficacy, if any, of eCODES is being evaluated in an ongoing clinical trial.

## Conclusions

We developed eCODES, a highly usable Web-based decision aid for surrogate decision makers of patients receiving prolonged mechanical ventilation, and then successfully implemented it in a clinical trial setting. Our experience shows that families of the critically ill desire electronic decision support and greatly prefer an electronic format to a printed alternative - even among the elderly who rated usability somewhat lower than younger participants. Electronic decision aids have great potential to contribute to more patient centered and family-orientated health care delivery in the very near future.

## Additional files

Additional file 1: Figure S1. Evolution of eCODES development. eCODES represents the third in a series of decision aid versions dating to 2010. Important failures and detours are shown that are noted here: Version 1 (app): we explored the expansion of decision aid v1 to a tablet computer/smartphone app, using its code as the foundation for programming. The app was successfully designed, but the development team dissolved at the end of the project leading to instability in app support and server maintenance, requiring us to abandon this angle. Another important limitation was that data could be transmitted from app to the server only, not from the server to the app. Version 2: However, we ultimately ended this partnership due to our concerns about the ability of the consultants to provide timely support and maintenance as well as a change in university regulation of electronic health data that prevented our use of a non-university server Additional file 2:
**Security standards of study data system used to present eCODES.**
Additional file 3:
**Instructions for usability testing.**
Additional file 4: Figure S2. Screenshots from eCODES compared to the original written decision aid. These are selected screenshots from the third and final version of the decision aid (eCODES) that are contrasted to the original material from the written decision aid.

## Abbreviations

eCODES: electronic Collaborative Decision Support
EHR: electronic health records
ICU: intensive care unit
ePRO/PRO: (Electronic) Patient-reported outcomes
ProVent: prolonged mechanical ventilation prediction model
