# Supplementary figures and images for: Development and usability testing of a Web-based decision aid for families of patients receiving prolonged mechanical ventilation
Source: Ann Intensive Care. 2015 Mar 25;5:6. doi: 10.1186/s13613-015-0045-0 (PMC4385299; doi:10.1186/s13613-015-0045-0)

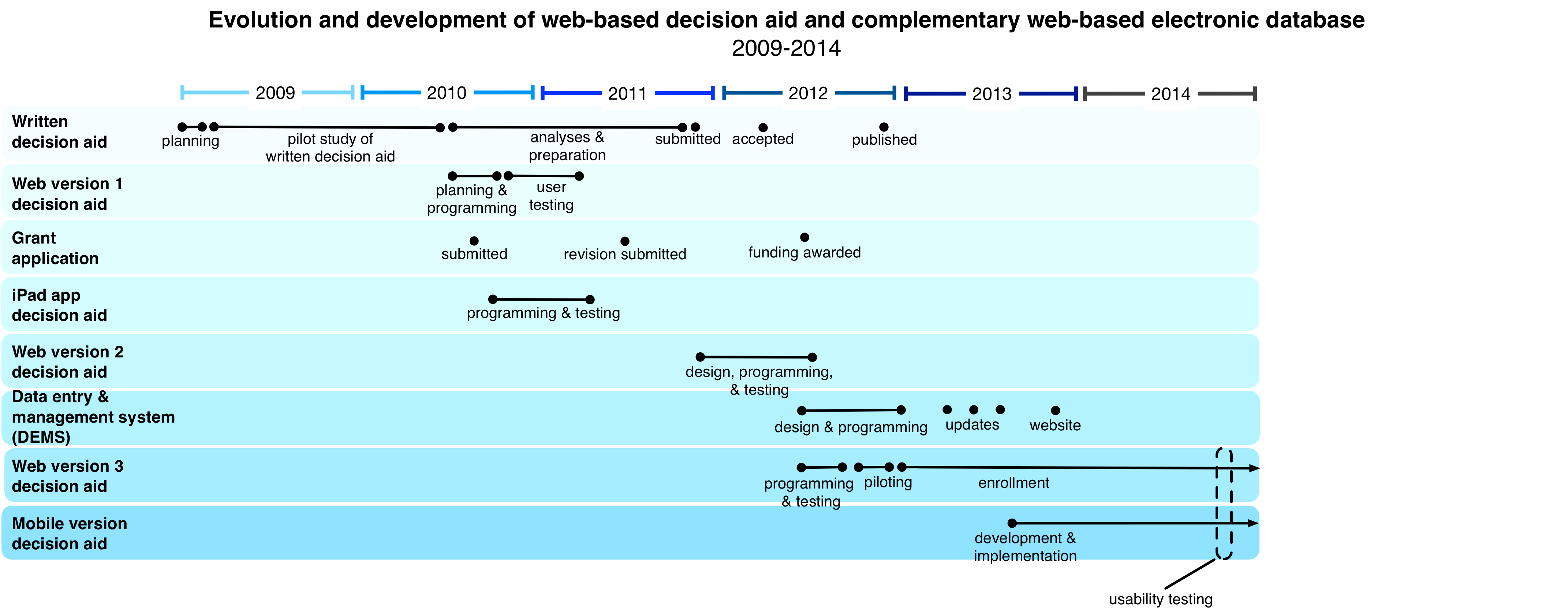

Supplement: Additional file 1: Figure S1. — Evolution of eCODES development. eCODES represents the third in a series of decision aid versions dating to 2010. Important failures and detours are shown that are noted here: Version 1 (app): we explored the expansion of decision aid v1 to a tablet computer/smartphone app, using its code as the foundation for programming. The app was successfully designed, but the development team dissolved at the end of the project leading to instability in app support and server maintenance, requiring us to abandon this angle. Another important limitation was that data could be transmitted from app to the server only, not from the server to the app. Version 2: However, we ultimately ended this partnership due to our concerns about the ability of the consultants to provide timely support and maintenance as well as a change in university regulation of electronic health data that prevented our use of a non-university server [file 13613_2015_45_MOESM1_ESM.png]

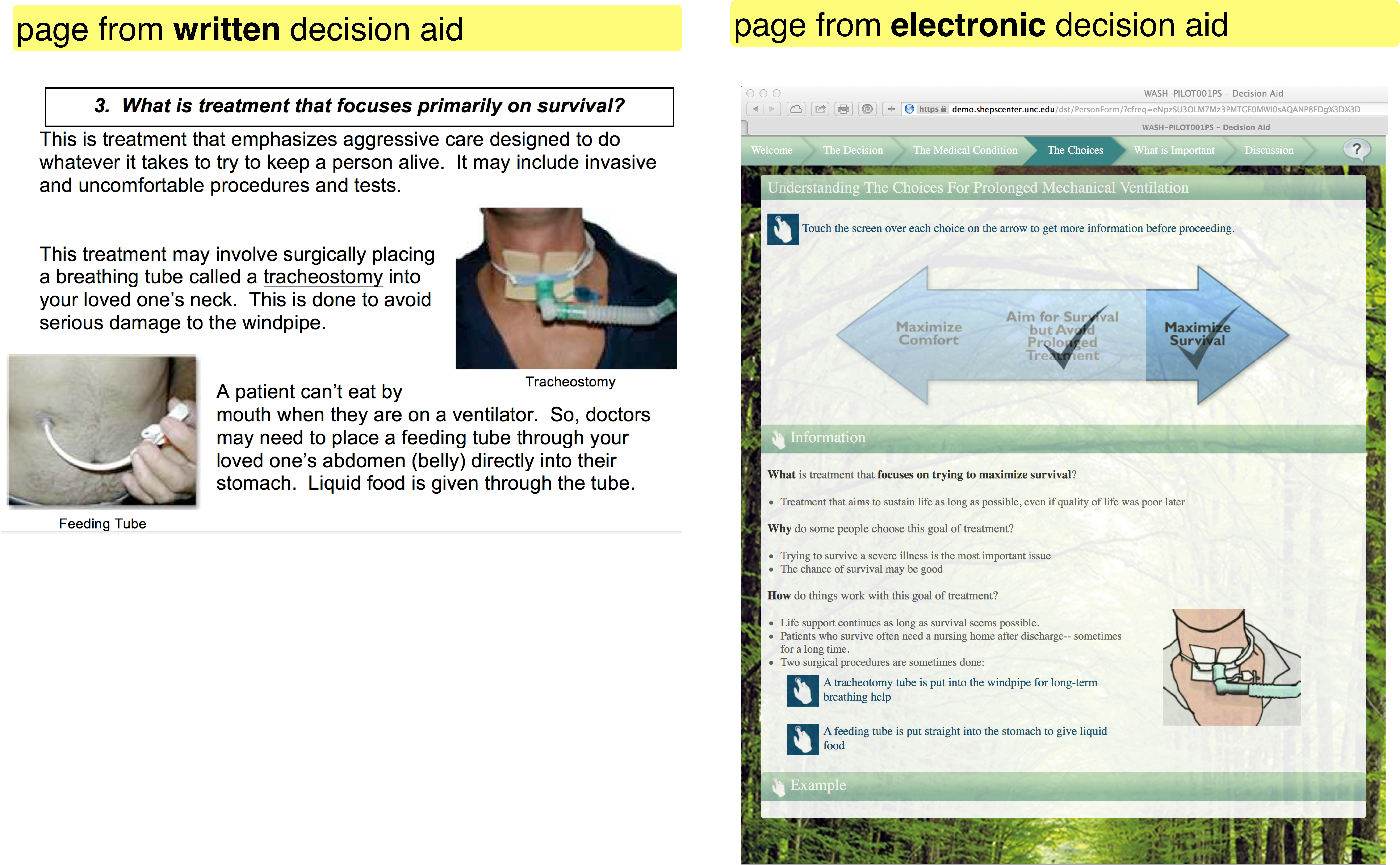

Supplement: Additional file 4: Figure S2. — Screenshots from eCODES compared to the original written decision aid. These are selected screenshots from the third and final version of the decision aid (eCODES) that are contrasted to the original material from the written decision aid. [file 13613_2015_45_MOESM4_ESM.png]
