# Supplementary material for: Development and usability testing of a Web-based decision aid for families of patients receiving prolonged mechanical ventilation
Source: Ann Intensive Care. 2015 Mar 25;5:6. doi: 10.1186/s13613-015-0045-0 (PMC4385299; doi:10.1186/s13613-015-0045-0)
Supplement: Additional file 2: — Security standards of study data system used to present eCODES. [file 13613_2015_45_MOESM2_ESM.docx]

**Online Supplement 1: Security standards of study data system used to present eCODES**

**1. Security standards and protocols used for web-based study development products**

Background: The study’s data entry and management system (DEMS) uses a MySQL database server, Apache web server, and a storage area network (SAN) file system. Many security standards for data storage, access, and server/network management were implemented to protect patient health information and patient identifying information. The appearance and functionality of the DEMS electronic questionnaire user interface was designed for touch-screen computers, simple navigation, and efficient data entry. This was achieved using a combination of programming techniques, which provide asynchronous data submission to the web server (AJAX) resulting in a fluid (no delay) user experience when navigating and interacting with the screens of an instrument.

Web Server: Secure interaction with the DEMS happens through an Apache web server utilizing Secure Socket Layers (SSL) to encrypt all data transmissions to and from the web browser.

Database: A MySQL database runs on a secure server. Access to this server is strictly limited to a handful of servers and named programmers.

File Storage: All files related to the DEMS are stored on a Storage Area Network (SAN). This means that files are distributed over a wide array of hardware. In most cases, if a hardware failure occurs, in/out (I/O) service will not be lost.

Server Housing and Management: All servers are housed in a secure data center managed by the University of North Carolina – Chapel Hill central information technology (IT) department. Their role is to monitor the hardware and provide consistent power, network access, and cooling. The Cecil G. Sheps Center System Administrators manage the applications on the servers (Apache, MySQL, etc.).

Server Redundancy: All servers are “virtualized” and can be easily migrated to different hardware as requirements change or hardware ages. If a hardware failure occurs, the server can be restored on different hardware within minutes.

User Authentication: All users and participants must authenticate to access data from the DEMS web interface. Users utilize a user ID and password combination to access the site upon which their permissions within the system are limited based on their assigned role. Participants utilize a personalized URL from an email and authenticate with their date of birth. Analytical programmers building reports connect statistical program output (e.g., SAS) directly to the database and use a read only user Id and password. Web/Database programmers connect directly to the database using a user ID and password.

Piloting: Before implementation in a clinical trial setting and after exhaustive reviews by study staff involving multiple rounds of hand-checking of web-entered data, this electronic questionnaire system was successfully piloted by administration to 5 patients and caregivers within a full study protocol in November 2013.
