# Supplementary material for: Development and usability testing of a Web-based decision aid for families of patients receiving prolonged mechanical ventilation
Source: Ann Intensive Care. 2015 Mar 25;5:6. doi: 10.1186/s13613-015-0045-0 (PMC4385299; doi:10.1186/s13613-015-0045-0)
Supplement: Additional file 3: — Instructions for usability testing. [file 13613_2015_45_MOESM3_ESM.docx]

**ONLINE SUPPLEMENT 2: Instructions for usability testing**

**PART 1**

**What is the situation?** Imagine that your loved one is sick in an intensive care unit (ICU). They have been on a life support machine (a mechanical ventilator) for over 10 days—much longer than average. They are 60 years old and have just started to need dialysis in the ICU because of kidney problems.

**What is the problem?** Your loved one is so sick that they can’t make decisions for themself. They need you to help make a decision about what to do next about life support.

**What could help you make a decision?** The computer program you are about to use is called a **decision aid**. It helps people think through a difficult decision.

**PART 2**

**What are you asking me to do now?**

1. We want you to look at this decision aid—again, imagining that you are in this situation.
2. You can answer the questions in the decision aid however you like.
3. Give feedback to us about the how usable the decision aid was to you. We are interested in your feedback on the decision aid computer program—not about how you answered questions in the program itself.

*Remember, this is an **imaginary situation**. It does not relate directly to any particular patient. We do not record any personal information. This is confidential!

Thanks for your time! Your feedback helps us to make better computer programs to help people in very difficult, stressful situations.
